# Mononuclear cell therapy of neonatal hypoxic‐ischemic encephalopathy in preclinical versus clinical studies: A systematic analysis of therapeutic efficacy and study design

**DOI:** 10.1002/nep3.29

**Published:** 2023-12-30

**Authors:** Alexander M. Scrutton, Francesca Ollis, Johannes Boltze

**Affiliations:** ^1^ School of Life Sciences University of Warwick Coventry UK; ^2^ Neurobiology Division, MRC Laboratory of Molecular Biology University of Cambridge Cambridge UK

**Keywords:** cell therapy, hypoxic‐ischemic encephalopathy, mononuclear cells, translational research, treatment efficacy

## Abstract

**Background:**

Hypoxic‐ischemic encephalopathy (HIE) is a devastating condition affecting around 8.5 in 1000 newborns globally. Therapeutic hypothermia (TH) can reduce mortality and, to a limited extent, disability after HIE. Nevertheless, there is a need for new and effective treatment strategies. Cell‐based treatments using mononuclear cells (MNCs), which can be sourced from umbilical cord blood, are currently being investigated. Despite promising preclinical results, there is currently no strong indicator for the clinical efficacy of the approach. This analysis aimed to provide potential explanations for this discrepancy.

**Methods:**

A systematic review and meta‐analysis were conducted according to the Preferred Reporting Items for Systematic Reviews and Meta‐Analysis guidelines. Preclinical and clinical studies were retrieved from PubMed, Web of Science, Scopus, and clinicaltrials.gov using a predefined search strategy. A total of 17 preclinical and 7 clinical studies were included. We analyzed overall MNC efficacy in preclinical trials, the methodological quality of preclinical trials, and relevant design features in preclinical versus clinical trials.

**Results:**

There was evidence for MNC therapeutic efficacy in preclinical models of HIE. The methodological quality of preclinical studies was not optimal, and statistical design quality was particularly poor. However, methodological quality was above the standard in other fields. There were significant differences in preclinical versus clinical study design including the use of TH as a baseline treatment (only in clinical studies) and much higher MNC doses being applied in preclinical studies.

**Conclusions:**

Based on the analyzed data, it is unlikely that therapeutic effect size is massively overestimated in preclinical studies. It is more plausible that the many design differences between preclinical and clinical trials are responsible for the so far lacking proof of the efficacy of MNC treatments in HIE. Additional preclinical and clinical research is required to optimize the application of MNC for experimental HIE treatment.

## Key points

Significant findings of the study

This study identified significant design differences between preclinical and clinical trials using mononuclear cells (MNCs) as an experimental hypoxic‐ischemic encephalopathy (HIE) treatment. These differences may explain the lack of clinical efficacy proof of MNC in HIE.

What this study adds to the advances in the areas of basic, translational, or clinical neuroscience

This is a systematic review and meta‐analysis investigating design differences in preclinical versus clinical studies using MNC for HIE. Identified differences, together with spotted shortcomings in preclinical methodological study quality, will improve future translational research in the field.

## Graphical abstract

Preclinical and clinical studies using mononuclear cell treatment for neonatal hypoxic‐ischemic encephalopathy were analyzed and compared in a systematic review with meta‐analysis. Clear therapeutic effects were reported in preclinical trials. Those beneficial effects were independent of major study design features and could not be recapitulated in clinical studies. While the clinical studies were primarily designed to detect safety, what may have contributed to this situation, there were also many differences in preclinical versus clinical study design. For instance, none of the preclinical studies employed therapeutic hypothermia as baseline treatment, what is an established standard in clinical trials. There were also remarkable differences between routes and time of cell administration. Moreover, most preclinical studies used xenogeneic cells, whereas most clinical studies expectedly used autologous cells. There were further study design differences. Additional analyses performed comprise, among others, preclinical study quality and the correlation of preclinical study quality and reported therapeutic effect size.

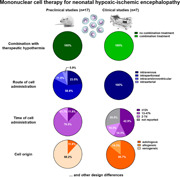

## 1 INTRODUCTION

Neonatal hypoxic‐ischemic encephalopathy (HIE) is a devastating condition resulting from perinatal asphyxia, leading to global cerebral hypoxia and ischemia. HIE is characterized by neurological dysfunction in neonates including, but not limited to, reduced levels of consciousness, seizures, challenges to initiate and maintain proper respiration, abnormal tone, and pathological reflexes. HIE is among the 10 leading causes of lost years of life worldwide.^1^ About 15%–60% of affected neonates do not survive HIE, and those who do typically experience sensorimotor deficits, mental retardation, and mental disability throughout their life. Thus, HIE exerts significant distress to patients and their relatives and causes a massive socioeconomic burden due to high lifetime treatment costs. Unfortunately, HIE is not a rare condition. It is estimated that it affects around 8.5 in 1000 live births.^2^ Although most cases (96%) are reported from low‐ and middle‐income countries, HIE is also a problem in high‐income nations, as exemplified by an incidence of 2.6 in 1000 live births in the United Kingdom.^3^

Therapeutic hypothermia (TH) has been established as a treatment for HIE and can reduce both mortality and disability in moderate to severe HIE.^4^, ^5^ However, overall effects are modest as most survivors still develop moderate or severe disability, and studies investigating long‐term (i.e., 6–7 years) outcomes have revealed inconsistent results regarding disability reduction.^6^ Thus, there is an urgent need for additional therapeutic strategies in HIE.

Cell‐based strategies are an emerging paradigm in the treatment of acute and chronic neurodegenerative diseases. Originally intended to replace injured brain tissue, it has become clear that therapeutically active cells mainly exert supportive mechanisms, so‐called “bystander effects” such as anti‐inflammation, antiapoptosis, and increased levels of angiogenesis.^7^ Whereas there is great promise in naïve and pluripotent stem cell populations, such as induced pluripotent stem cells, adult cell populations are currently easier and, to some extent, safer to use in a clinical setting. The safety of adult cell populations is mainly determined by their limited proliferation and differentiation capability, dramatically reducing the risk of tumorigenesis. They can also be obtained autologously, so their application does not require immunosuppressive treatments and therefore avoids potential immunological complications arising during treatment. Some adult cell populations such as mesenchymal stem cells (MSC)^8^ can also avoid immune responses and may even be safe to use without immunosuppression in an allogeneic setting.

Mononuclear cells (MNCs) are typically obtained from human umbilical cord blood (hUCB) or bone marrow (BM). MNCs are mixed cell populations containing mature cells and between 0.5% and 3% of hematopoietic stem and progenitor cells. BM and hUCB MNC have been shown to effectively mitigate the consequences of acute focal cerebral ischemia (stroke) in animal models,^9^, ^10^ have a therapeutic time window of about 3 days,^11^, ^12^ and are compatible with as well as beneficial after thrombolysis.^13^ MNCs are among the most frequently used cells in clinical trials focusing on stroke, together with MSC. Whereas MSCs are safely applicable and have excellent therapeutic potential, the generation of sufficient amounts of autologous MSCs takes a considerable amount of time. Due to their size, MSC use may also pose some risk of microembolism after both intravenous and intraarterial administration, when conducted improperly.^14^, ^15^ There are no such risks when using the smaller MNC. Moreover, MNC can directly interact with host cells to exert therapeutic effects in numerous conditions.^16^, ^17^

Consequently, MNC‐based interventions have been frequently investigated regarding their therapeutic potential in HIE. Clinical trials have been conducted after promising initial results were reported from preclinical studies using MNC in HIE animal models.^18^, ^19^ However, mixed results were reported from these clinical trials^20^ as therapeutic effects could not be confirmed or massively fell short of what was reported in animal studies. The reasons for this translational failure are not well understood, but there are in principle, two possible explanations. The first explanation would be that the therapeutic impact of MNC may be sufficient to induce beneficial effects after a limited, that is, focal ischemic event (stroke) but may not be strong enough to do so after a global hypoxic‐ischemic event with widespread brain damage and potentially catastrophic impact on brain function. The second explanation is that preclinical and clinical studies have fundamental differences in their design which may contribute to the observed translational failure.

Indeed, it has been shown that there are significant differences in key study design parameters in preclinical versus clinical studies on cell‐based therapies in stroke and that these differences may explain the lack of successfully translating promising preclinical results into clinical treatments.^21^ The situation may be similar in HIE, but a systematic analysis of preclinical versus clinical approaches is lacking so far.

Thus, this study had two major aims. The first was to systematically analyze the efficacy of MNC therapies for HIE in preclinical studies to assess their therapeutic impact. By considering different study design parameters such as timing or route of MNC administration, and identifying potential relationships between therapeutic efficacy, this may help inform approaches in future clinical trials. The second aim was to compare the design of preclinical versus clinical studies using MNC‐based interventions for HIE. We also assessed the methodological quality of preclinical studies using MNC populations for experimental HIE treatment. To the best of our knowledge, this is the first systematic review and meta‐analysis addressing these aims.

## 2 METHODS

### 2.1 Approach and search strategy

We performed a systematic review and meta‐analysis to obtain relevant data from published literature and databases. The systematic review was carried out according to the Preferred Reporting Items for Systematic Reviews and Meta‐Analysis (PRISMA) guidelines.^22^ Preclinical and clinical studies using MNC therapy for neonatal HIE and published before January 1, 2023 were identified in PubMed, Web of Science, Scopus, and clinicaltrials.gov (the latter for clinical studies only) using a set of predefined search terms. Search terms were organized in “columns” and connected with the Boolean logical operator OR within individual columns. Columns were connected by the logical operator AND (please see Tables S1 and S2 for details). When searching clinicaltrials.gov, terms not describing the condition were entered in the “other terms” or “intervention/treatment” field. All identified database entries were collected, and duplicates were removed. A three‐step selection strategy was applied. First, all titles were screened for relevancy. Second, abstracts (where available) were considered. Thereafter, a full‐text assessment was performed as the third step. Predefined exclusion and inclusion criteria (see below) were applied. The literature search was performed by two investigators (A. M. S. and F. O.). In case of doubt regarding study eligibility, the senior investigator (J. B.) was consulted for clarification.

### 2.2 Inclusion and exclusion criteria

We included all studies on neonatal/perinatal HIE applying a cell treatment using MNC. Studies using animal models of HIE, clinical trials, and clinical case studies were eligible for inclusion.

We excluded studies not focusing on neonatal/perinatal HIE (e.g., other conditions such as focal cerebral ischemia/stroke, traumatic brain injury, noncerebral ischemia, tumors, adult HIE, and in vitro studies), studies that did not include a cell treatment, and cell treatment studies using cell populations other than MNC, including MNC‐derived populations such as CD34^+^ cells. Further, reviews and meta‐analyses were excluded, but their reference lists were screened for potentially eligible studies not identified by our search strategy. Studies published in languages other than English were also excluded. If no full text was accessible, the corresponding author of the respective paper was contacted via email, requesting an electronic copy. In case there was no reply even after sending a reminder, the study was excluded.

### 2.3 Assessment of preclinical study quality

Preclinical study quality was assessed using a quality score. The quality score was designed based on the 2009 Stroke Treatment Academic Round Table (STAIR^23^) as well as the 2009 and 2011 Stem Cell Therapies as an Emerging Paradigm in Stroke (STEPS^24^, ^25^) recommendations due to a lack of similar recommendations for the HIE field. The quality score comprised four different categories: adherence to general quality assurance criteria (category 1), animal model information (category 2), cell treatment information (category 3), and statistical design quality (category 4). Each category contained four individual items (Table 1).

**Table 1:** Quality assessment score of preclinical study on neonatal/perinatal HIE using MNC.

Item	Reported/not reported
Category 1: Adherence to General Quality Assurance Criteria	
Allocation concealment	1/0
Randomization	1/0
Blinded outcome assessment	1/0
Animal inclusion/exclusion criteria^a^	1/0
Category 2: Animal Model Information	
Defined species	1/0
Defined sex	1/0
Defined age	1/0
Physiological parameter monitoring (model induction)^b^	1/0
Category 3: Cell Treatment Information	
Cell characterization	1/0
Cell number or dose (range)	1/0
Cell viability check before transplantation	1/0
Cell fate after transplantation	1/0
Category 4: Statistical Design Quality	
Primary/secondary endpoint definition	1/0
A priori power analysis	1/0
A priori sample size calculation	1/0
Appropriate statistical test applied^c^	1/0

Abbreviations: HIE, hypoxic‐ischemic encephalopathy; MNC, mononuclear cell.

^a^ At least core body temperature should have been monitored and kept at/in a predefined level/range for assigning a score point.

^b^ At least one inclusion or exclusion criterion should have been reported for assigning a score point.

^c^ A score point was assigned when parametric or nonparametric tests were selected after investigation of normal data distribution and when adequate tests for multiple comparisons including specified post hoc tests were selected (where applicable).

A score point was assigned when information regarding the respective item was reported. Total quality scores and scores for each individual category were then calculated.

### 2.4 Effect size calculation

Effect sizes were calculated on primary endpoints of preclinical studies which met the predefined inclusion and none of the exclusion criteria. In case primary endpoints were not defined by the authors, a ranking system was applied for definition. Any functional outcome was ranked first, followed by lesion volumetry/brain atrophy, histological endpoints, and molecular biology endpoints, in that order. The highest‐ranked endpoint in a study was defined as the primary endpoint. In case multiple time points or cell doses were investigated, the effect size was calculated for the latest time point and the highest cell dose, respectively. Hedge's *g*, a well‐established unit to measure the effect size for the difference between means, was calculated by obtaining sample sizes, standard deviations, and means from each respective study. WebPlotDigitalizer 4.4 was used to obtain accurate values from figures in cases where they were not provided in the text, figure legends, or supplementary material. Due to the relatively small sample size in the studies that were included in the meta‐analysis (<20), we computed Hedge's *g* with bias correction.

### 2.5 Risk of bias assessment

All preclinical studies were investigated for potential risks of bias in five predefined categories related to the quality score assessment previously conducted: selection bias (allocation concealment and randomization), performance bias (blinding of outcome assessment), detection bias (predefined endpoints), reporting bias (appropriate use of statistical tests), and attrition bias (incomplete outcome data). The studies' respective results in each risk of bias domain were used to formulate an overall bias assessment, which remained consistent throughout.

### 2.6 Statistics and meta‐analysis

Both preclinical and clinical studies were comprehensively analyzed to obtain important information regarding individual study characteristics. This was then used to conduct a stratified meta‐analysis comparing effect sizes of individual studies. Before statistical testing, MNC effect size data was tested for normality using the D'Agostino and Pearson test, the Shapiro–Wilk test, and QQ plotting. Since the data was not normally distributed, nonparametric tests were used throughout the analysis. Unless otherwise stated, a Kruskal–Wallis test with Dunn's multiple comparisons was used for groupwise comparisons. The Spearman rank‐order correlation coefficient was used as a nonparametric measure for correlation analysis. We considered a *p* value of less than 0.05 to be statistically significant, and statistical significance is indicated by *p ≤ 0.05 and **p ≤ 0.01. A trend was identified in the case that 0.05 ≤ *p* < 0.1. All data was analyzed on GraphPad Prism 9.5.1. Data is shown as mean ± SD unless otherwise stated.

## 3 RESULTS

### 3.1 Literature search

**Figure 1:**
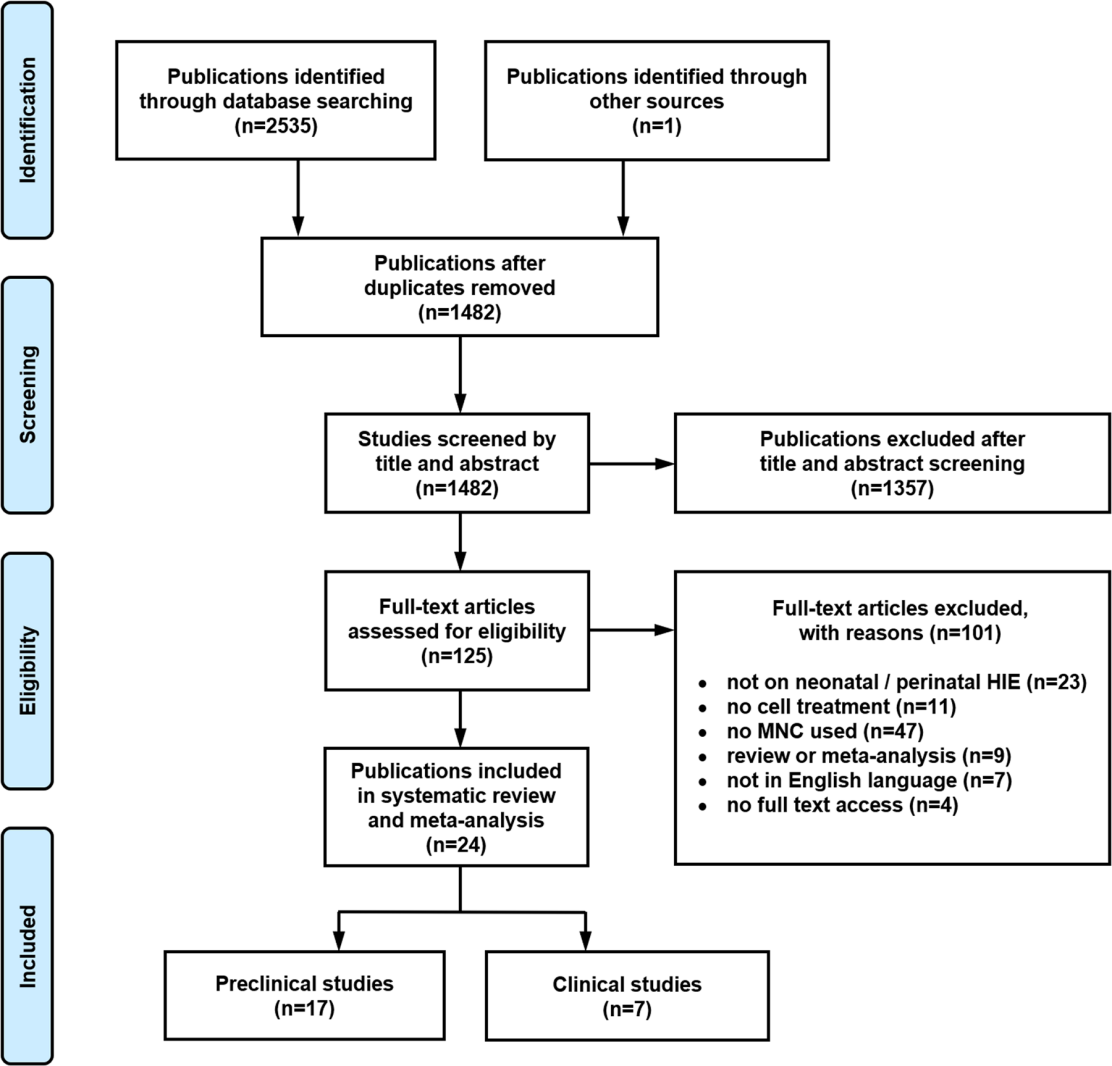
Systematic literature search flow diagram. The diagram presents the number of studies processed according to the Preferred Reporting Items for Systematic Reviews and Meta‐Analysis guidelines. A total of 17 preclinical and 7 clinical studies were included in the systematic review and meta‐analysis. HIE, hypoxic‐ischemic encephalopathy; MNC, mononuclear cell.

The literature search revealed a total of 2535 database entries which were reduced to 1482 after duplicate removal. Screening by title and abstract excluded 1357 studies. One additional paper was retrieved from another source (a reference list). The full text of the remaining 125 studies was accessed, and 24 studies (17 preclinical and 7 clinical studies) were finally included in the systematic review and meta‐analysis. The PRISMA flowchart is presented in Figure 1, which also provides additional details on reasons for study exclusion during the full‐text assessment. Detailed characteristics of included preclinical studies are provided in Table S3, and those of included clinical studies are provided in Table S4.

### 3.2 Robust efficacy of MNC therapy in preclinical HIE studies

**Figure 2:**
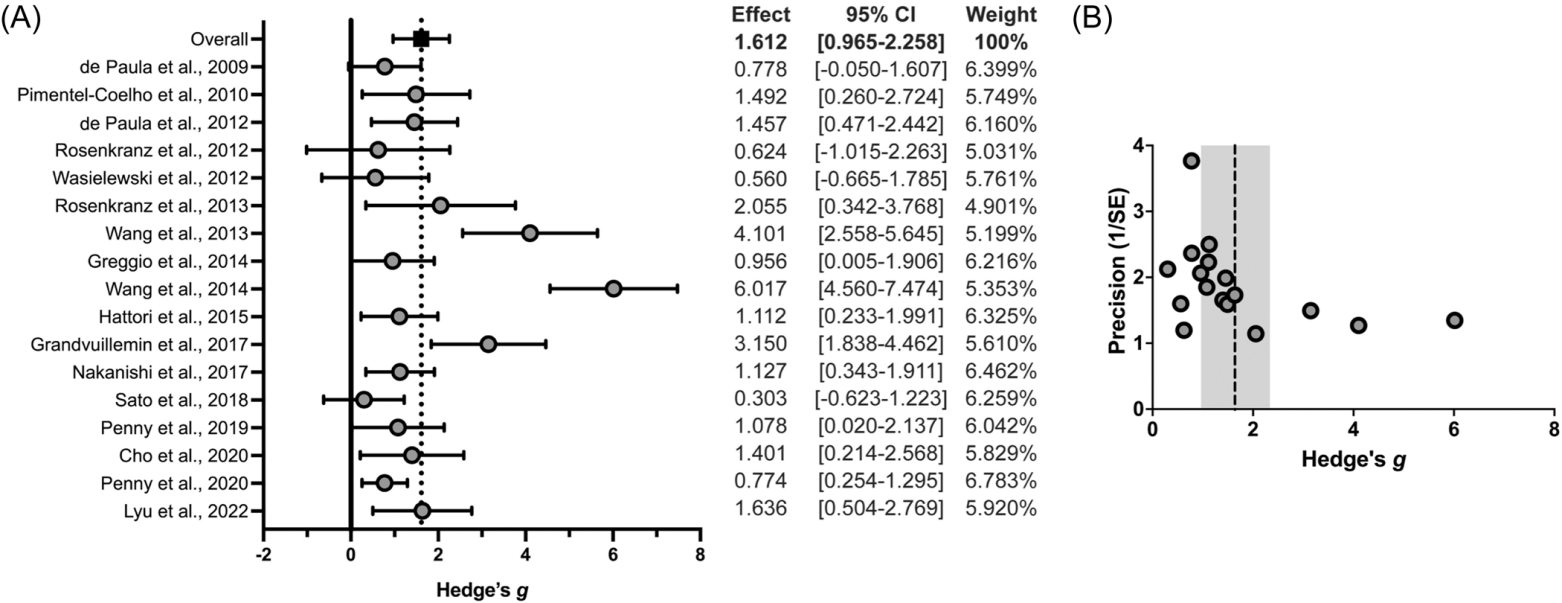
Functional and structural outcomes of mononuclear cell (MNC) treatment in preclinical trials. (A) Forest plot showing the effect sizes (Hedge's *g*), 95% confidence intervals (CIs), and relative weighting of preclinical trials assessing the therapeutic potential of MNC treatment for hypoxic‐ischemic encephalopathy in preclinical studies. Individual studies are indicated by gray circles, while the overall effect size is indicated by the dotted black line and black box. Data is shown as mean ± 95% CI. (B) Funnel plot showing effect sizes (gray circles) compared to their relative precision (1/SE). 95% of CIs are shown in gray.

Analysis of published preclinical data confirmed that transplantation of MNCs significantly improved outcomes in experimental models of HIE (Figure 2A). Statistically significant interstudy heterogeneities (*I* ^2^) were observed (78.6%; 95% confidence interval, CI [66.4–86.4]) and were accounted for using a random effects model. The pooled effect size of the published data was 1.612 (95% CI [0.965–2.258]) and significantly favored MNC intervention (Figure 2A; *z* = 4.88; *p* < 0.01). Two studies in the meta‐analysis exhibited an exaggerated effect size^26^, ^27^ but a leave‐one‐out analysis for both studies retained a statistically significant benefit of MNC treatment (data not shown). Moreover, the studies were investigated for the presence of publication bias. Funnel plotting suggested a moderate right‐side bias, but Egger's regression test revealed that this was not statistically significant (Figure 2B; *R* ^2^ = −0.028; *F* = 0.569; *p* = 0.462).

### 3.3 The preclinical efficacy of MNC therapy is not determined by study design parameters

**Figure 3:**
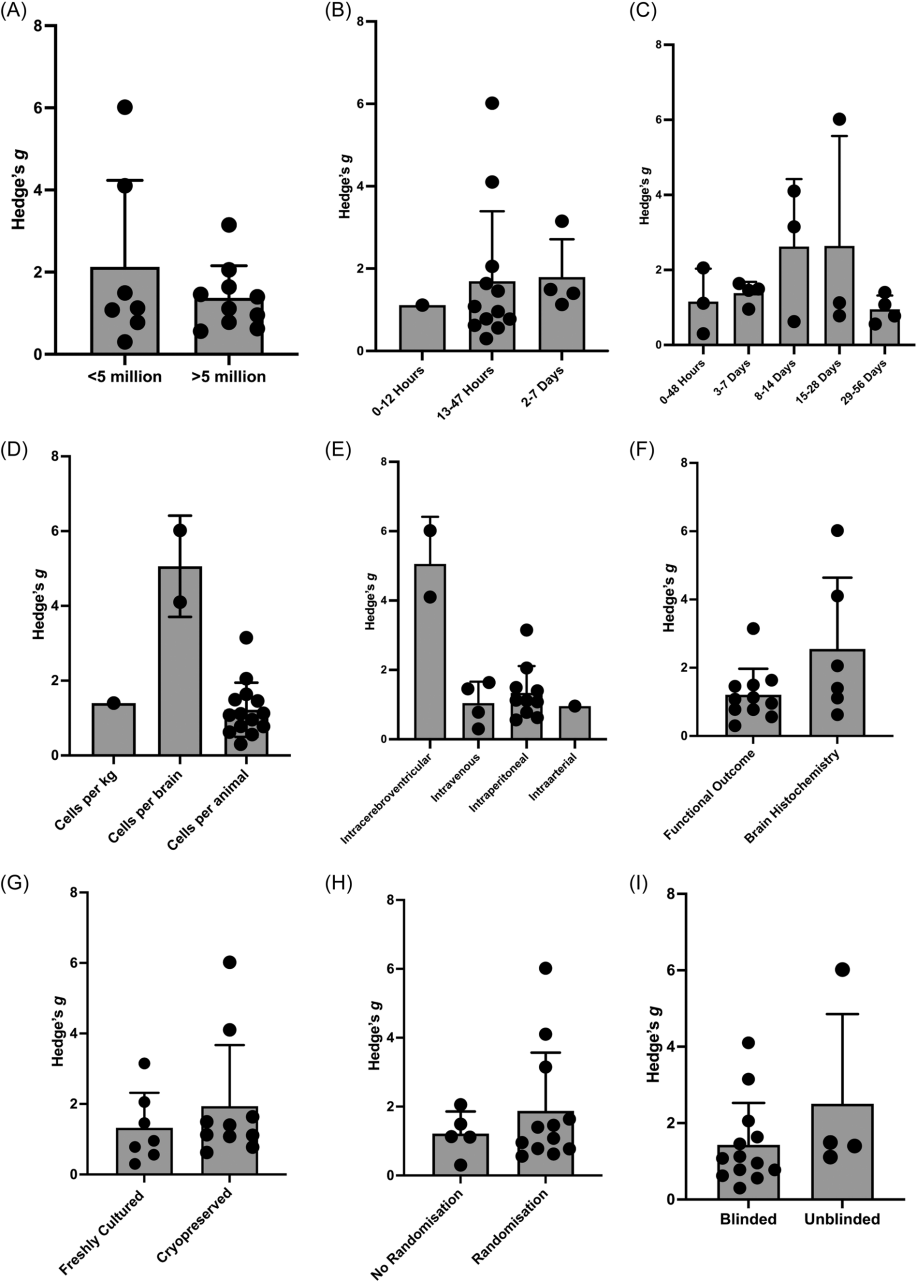
Preclinical study design parameters do not determine MNC treatment efficacy. (A) Effect sizes in preclinical studies compared to reported cell dosage, independent of dosing paradigm. (B) Effect sizes in preclinical studies compared to the reported time of MNC administration. (C) Effect sizes in preclinical studies compared to reported endpoint measurement time. (D) Effect sizes in preclinical studies compared to reported dosing paradigm. (E) Effect sizes in preclinical studies compared to the reported route of MNC administration. (F) Effect sizes in preclinical studies compared to reported endpoint measurement type. (G) Effect sizes in preclinical studies depend on whether MNCs were cryopreserved before transplantation. (H) Effect sizes in preclinical studies depending on whether randomization was applied. (I) Effect sizes in preclinical studies depend on whether a blinded outcome assessment was applied. Data are shown as mean ± standard deviation. MNC, mononuclear cell.

We performed a subgroup analysis in the published data from preclinical studies to determine the effects of different study design parameters on MNC treatment efficacy. Data was assessed for normality using the D'Agostino–Pearson and Shapiro–Wilk tests. The pooled MNC effect size data was not normally distributed (*K* ^2^ = 16.7, *p* < .01; *W* = 0.759, *p* < .01), and nonparametric tests were used for subsequent subgroup analysis. A variety of study design parameters were analyzed to determine their relationship with the reported efficacy in preclinical studies. There were no statistically significant differences in effect sizes regarding MNC dosage (Figure 3A), regarding the time point of MNC administration (Figure 3B), or regarding how long after HIE induction the last endpoint measurement was reported (Figure 3C). There was a statistically significant difference in dosing paradigms (Figure 3D; *H*(2) = 5.17; *p* < 0.05). Studies that used “cells per brain” reported higher effect sizes than “cells per kg” and “cells per animal”. However, pairwise comparisons using Dunn's test revealed that individual differences between “cells per brain” and other groups were not statistically significant (Figure 3D), presumably due to the small sample size. Other study design parameters that were analyzed included route of administration (Figure 3E), endpoint measurement type (Figure 3F), present or absent cryopreservation before MNC administration (Figure 3G), randomization of HIE animals into treatment and control groups (Figure 3H) and blinded versus unblinded endpoint assessment (Figure 3I). None of these study design parameters statistically significantly impacted the efficacy of MNC treatment in the analyzed preclinical studies. Moreover, no trends (*p* > 0.05 and *p* ≤ 0.1) were observed.

### 3.4 Suboptimal use of quality assurance criteria and poor statistical design quality in preclinical studies

**Figure 4:**
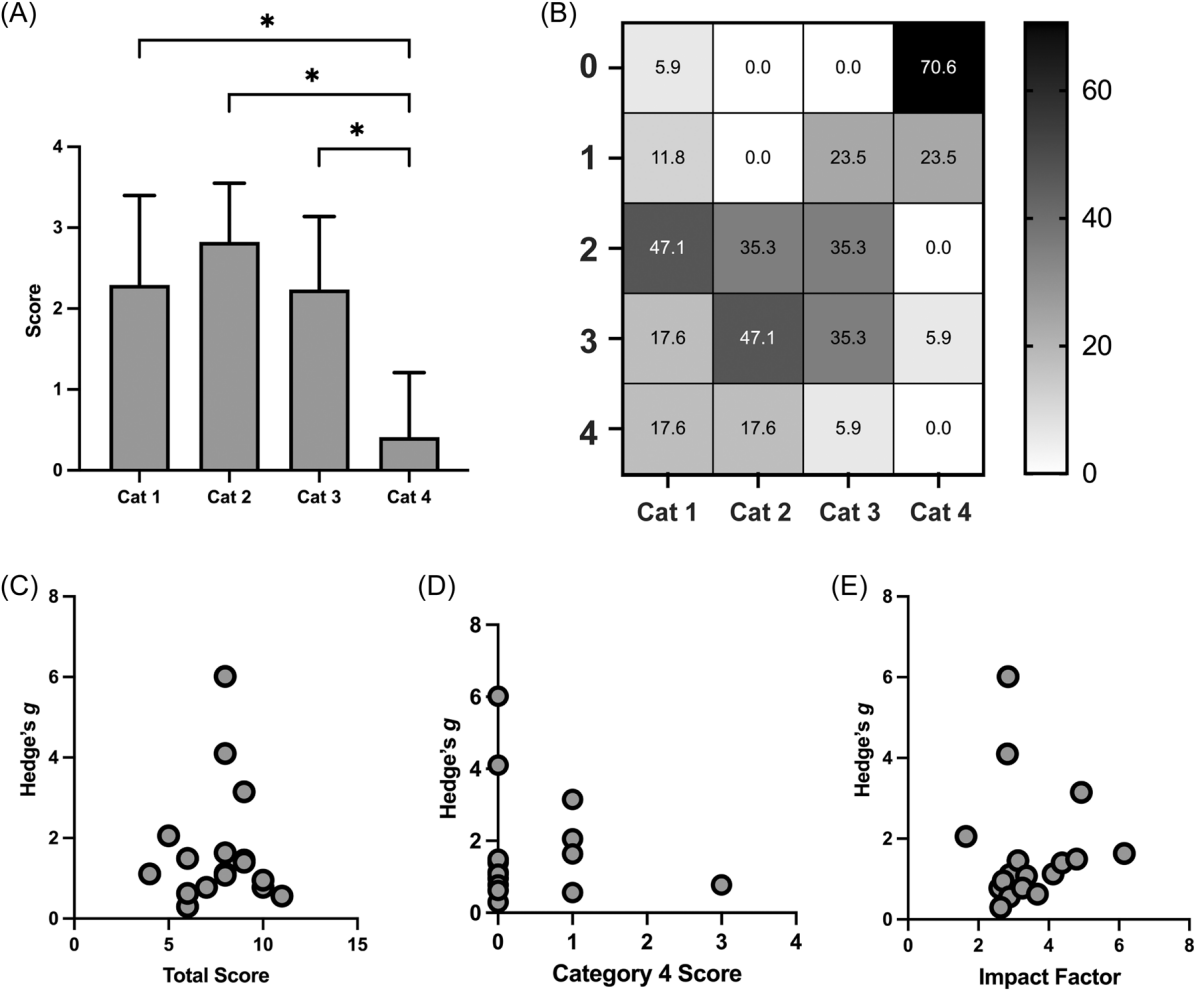
Suboptimal use of quality assurance criteria and poor statistical design quality in preclinical studies. (A) Quality scores of preclinical studies in individual quality score categories. The quality scoring system consisted of four categories (category 1: adherence to quality assurance criteria; category 2: animal model information; category 3: cell treatment information; category 4: statistical design quality), each with a maximum score of 4. (B) Frequency (%; greyscale) of quality scores of preclinical studies in individual categories (within boxes). Categories 1–4 are shown left to right. Individual scores (1–4) are shown from top to bottom. (C) Correlation between overall quality scores in preclinical studies in their respective effect sizes. (D) Correlation between category 4 quality scores in preclinical studies in their respective effect sizes. (E) Effect sizes of preclinical studies compared to journal impact factor in the year of the study's publication. Data in (A) is shown as mean ± standard deviation.

A quality score analysis was conducted on published preclinical studies (Figure 4). The quality scoring system consisted of four categories (adherence to quality assurance criteria; animal model information; treatment information; statistical design quality; Table 1), each with a maximum score of four. Overall, the median quality score was 8 out of 16 (interquartile range, IQR 6–9), and the average quality score was 7.765. Average scores for individual categories were 2.294 (category 1), 2.824 (category 2), 2.235 (category 3), and 0.412 (category 4). All studies (100%) reported the age of the animals used, and the MNC dosage applied for experimental HIE treatment. Twelve studies (70.6%) reported randomization. Monitoring of physiological parameters was reported in four studies (23.5%). Only one study (5.9%) reported using a priori sample size calculation, and one study (5.9%) conducted an a priori power analysis. None of the studies defined primary and/or secondary endpoints. A detailed overview of the frequency of application of individual quality score items is given in Table 2.

**Table 2:** Frequency of application of individual quality score items.

Item	Reported in (%) of studies
Category 1: Adherence to General Quality Assurance Criteria	
Allocation concealment	52.90
Randomization	70.60
Blinded outcome assessment	76.50
Animal inclusion/exclusion criteria	29.40
Category 2: Animal Model Information	
Defined species	94.10
Defined sex	64.70
Defined age	100.0
Physiological parameter monitoring (model induction)	23.5
Category 3: Cell Treatment Information	
Cell characterization	23.5
Cell number or dose (range)	100.0
Cell viability check before transplantation	47.1
Cell fate after transplantation	52.9
Category 4: Statistical Design Quality	
Primary/secondary endpoint definition	0.0
A priori power analysis	5.9
A priori sample size calculation	5.9
Appropriate statistical test applied	29.4

Studies had statistically significant lower quality scores in category 4 (statistical design quality) compared to other categories (Figure 4A; *p* < .01). Indeed, the preclinical data set showed a lack of sufficient statistical design quality with 12 studies (70.6%) not receiving a single score point in this category (Figure 4B). The median quality score for category four was 0 (IQR: 0–1), with an average score of 0.41. No study achieved the maximum possible score (Figure 4B). There was a formal weak negative correlation between effect sizes and overall quality score (Figure 4C; *r*(15) = −0.06; *p* = .83). However, this was not statistically significant, and the *p* value was sufficiently high to accept the null hypothesis. This suggests that there was no relationship between overall quality scores and preclinical effect sizes. There was also a formal weak positive correlation between the effect size and the quality scores of category 4 (Figure 4D; *r*(15) = 0.07), but this was also not statistically significant (*p* = 0.80). Thus, preclinical studies had consistently low scores in applying sufficient statistical quality assurance methods, but this was not a major determinant of the heterogeneity in effect sizes between these studies.

### 3.5 Effect size versus journal impact factor

The impact factor of the journal in the year of publication was not correlated to the therapeutic effect size reported in the respective study (Figure 4E), indicating that the most prominent indicator of journal quality has no influence on reported effect sizes. Interestingly, the studies reporting the highest effect sizes (6.0 and 4.1, respectively) were published in a journal with a comparatively low impact factor (about 2.8).

### 3.6 Risk of bias assessment

Presumably due to the suboptimal implementation of quality assurance methods, many of the preclinical studies had a high risk of bias (Table 3; red circles), and 15 out of 17 studies had at least moderate concern of bias (Table 3; yellow circles). Only 3 out of 17 studies had no high concern of bias in any of the assessed domains. Studies consistently had a particularly high degree of bias in domains 3 and 4 (Table 3; D3, D4), suggesting that bias in detection bias (predefined endpoints), and reporting bias (appropriate use of statistical tests were largely responsible for the high risk of bias that was observed.

**Table 3:** Risk of bias assessment for preclinical studies.

Preclinical studies	Risk of bias					
	D1	D2	D3	D4	D5	Overall
de Paula et al.^28^	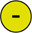	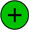	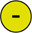	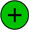	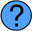	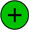
Pimentel‐Coelho et al.^29^	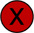	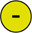	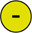	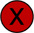	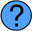	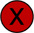
de Paula et al.^30^	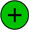	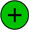	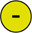	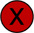	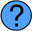	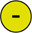
Rosenkranz et al.^19^	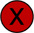	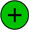	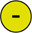	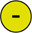	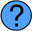	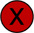
Wasielewski et al.^31^	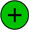	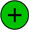	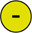	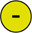	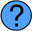	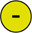
Rosenkranz et al.^32^	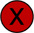	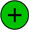	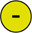	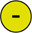	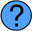	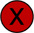
Wang et al.^26^	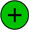	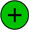	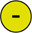	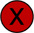	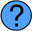	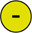
Greggio et al.^33^	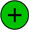	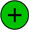	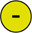	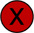	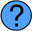	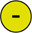
Wang et al.^34^	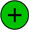	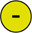	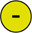	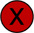	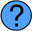	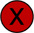
Hattori et al.^35^	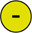	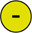	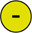	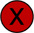	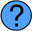	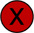
Grandvuillemin et al.^18^	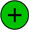	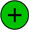	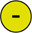	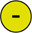	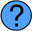	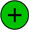
Nakanishi et al.^36^	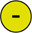	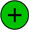	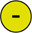	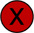	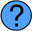	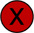
Sato et al.^37^	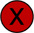	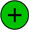	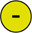	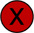	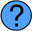	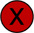
Penny et al.^38^	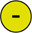	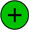	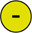	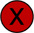	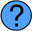	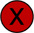
Cho et al.^39^	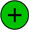	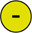	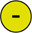	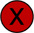	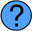	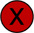
Penny et al.^40^	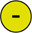	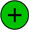	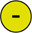	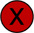	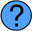	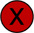
Lyu et al.^41^	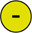	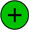	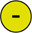	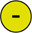	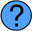	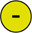

*Note*: Analysis was conducted in a total of five risks of bias domains, including selection bias (D1), performance bias (D2), detection bias (D3), reporting bias (D4), attrition bias (D5), and overall bias. Low (green circles), some (yellow circles), and high (red circles) concerns for bias, as well as no information (blue circles) are indicated.

### 3.7 Design differences between preclinical and clinical studies

While MNC therapy significantly improves functional and structural outcomes in preclinical HIE models (Figure 2), the efficacy of therapeutic intervention in clinical studies is less encouraging. We, therefore, also investigated potential design differences between preclinical and clinical studies. Several remarkable differences were identified.

#### 3.7.1 Subject age, combination therapy versus monotreatment and route of cell administration

First, clinical trial participants were consistently younger than their animal counterparts when considering the time of birth, not the developmental age. In 85.7% (*n* = 6) of clinical studies, participants were 3 days old or younger, while all subjects investigated in preclinical studies were at least 7 days old. Moreover, preclinical studies defined the age of their animals specifically, whereas clinical trials predominantly reported participants within an age range, so that determination of the exact age of clinical trial participants could not always be determined.

**Figure 5:**
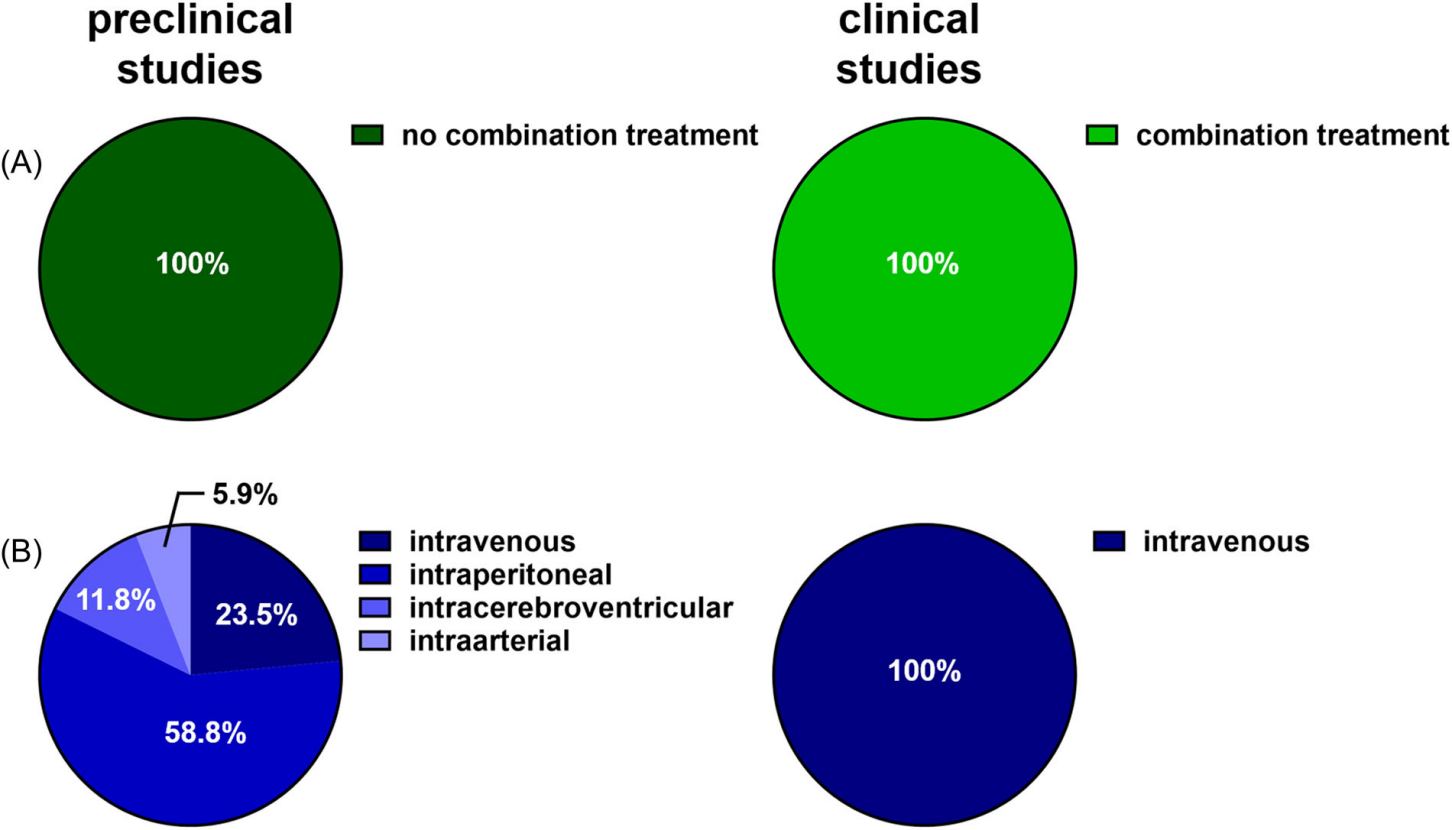
Combination therapy versus monotreatment and route of cell administration. (A) Proportion of studies using combination treatment versus no combination treatment. One preclinical study used a second cell population (allogeneic endothelial progenitor cells). The clinically applied combination treatment was therapeutic hypothermia. (B) The proportion of studies using respective routes of administration.

All (100%, *n* = 7) clinical studies applied a combination therapy approach, that is, TH together with MNC treatment. Contrastingly, none of the preclinical studies used TH or any other kind of combination treatment, although a co‐transplantation of allogeneic endothelial progenitor cells was reported in one preclinical study (Figure 5A). There were also clear differences in the route of MNC delivery between preclinical and clinical studies. A hundred percent (*n* = 7) of clinical studies used intravenous MNC injection, compared to only 23.5% (*n* = 4) of the preclinical studies. Instead, intraperitoneal injection was predominantly employed (58.8%, *n* = 10) in preclinical trials. This is comparable to intravenous administration in rodent models and was probably chosen due to animal size at the time point of treatment, making intravenous administration technically challenging. Other methods used were intracerebroventricular (11.8%; *n* = 2) and intraarterial injection (5.9%, *n* = 1) of MNC (Figure 5B). Thus, investigated routes of MNC transplantation are more diverse in preclinical studies.

#### 3.7.2 MNC dosing

**Figure 6:**
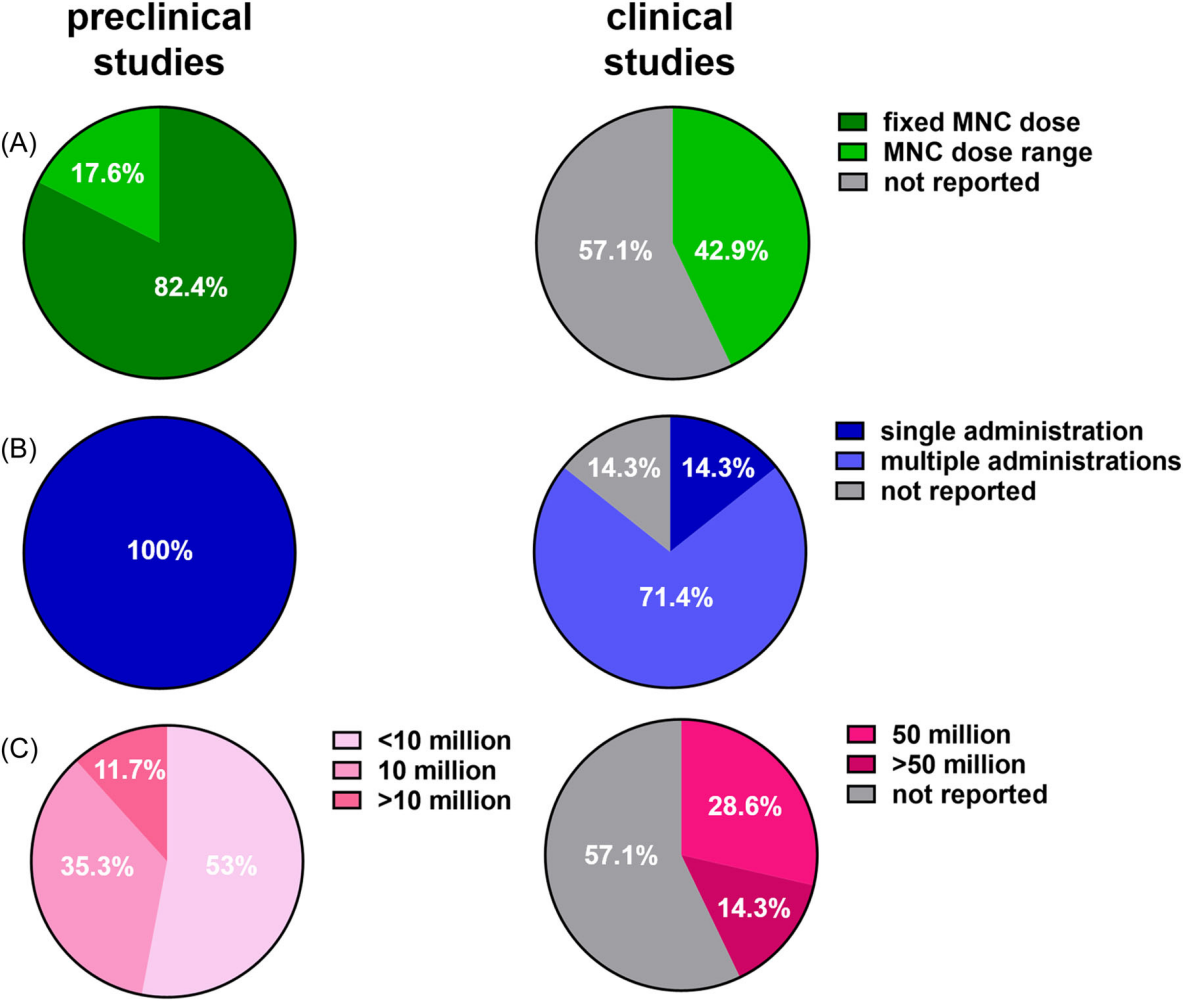
MNC dosing in preclinical versus clinical trials. (A) The proportion of studies using an MNC fixed versus dose range. (B) The proportion of studies using single versus multiple administrations/injections. (C) Absolute cell numbers transplanted in preclinical versus clinical trials. Note the considerable frequency of nonreporting in clinical studies. MNC, mononuclear cell.

Moreover, we found considerable differences in the MNC dosing approach used. A range of cells were injected in 42.9% of clinical trials (*n* = 3) whereas only 17.6% (*n* = 3) preclinical studies were working with a range of MNC numbers to be administered. Interestingly, 57.1% (*n* = 4) of the clinical studies did not report whether they injected a fixed dose or were working with a range of MNC numbers (Figure 6A). There were also differences regarding the dosing paradigm. 82.4% (*n* = 14) of preclinical studies injected a fixed dose of cells per individual, including the *n* = 2 studies (11.8%) conducting intracerebroventricular MNC injection. In contrast, in no clinical study, a fixed number of cells or cells/kg was injected while a range of MNC was injected per dose in 42.9% (*n* = 3) of clinical studies. All other studies (*n* = 4, 57.1%) did not report these aspects. While 100% (*n* = 17) of the preclinical trials relied on a single MNC dose, multiple doses of MNC were administered in 71.4% (*n* = 5) of the clinical trials. A single dose of cells was administered in 14.3% (*n* = 1) of the clinical studies, and one study (14.3%) did not report this (Figure 6B). Clinical studies consistently used higher absolute numbers of MNCs for treatment. Clinical studies reported treating with a minimum of 50 million cells, all administered in multiple doses (42.9%, *n* = 3), all other studies (57.1%, *n* = 4) did not report this. In contrast, 53% (*n* = 9) of the preclinical trials reported treating with less than 10 million cells/animal, exactly 10 million cells were administered in 35.3% (*n* = 6) of preclinical studies, and only 11.7% (*n* = 2) of the preclinical studies treated with more than 10 million (either cells/kg or cells/animal; Figure 6C).

#### 3.7.3 Timing of administration and MNC origin

**Figure 7:**
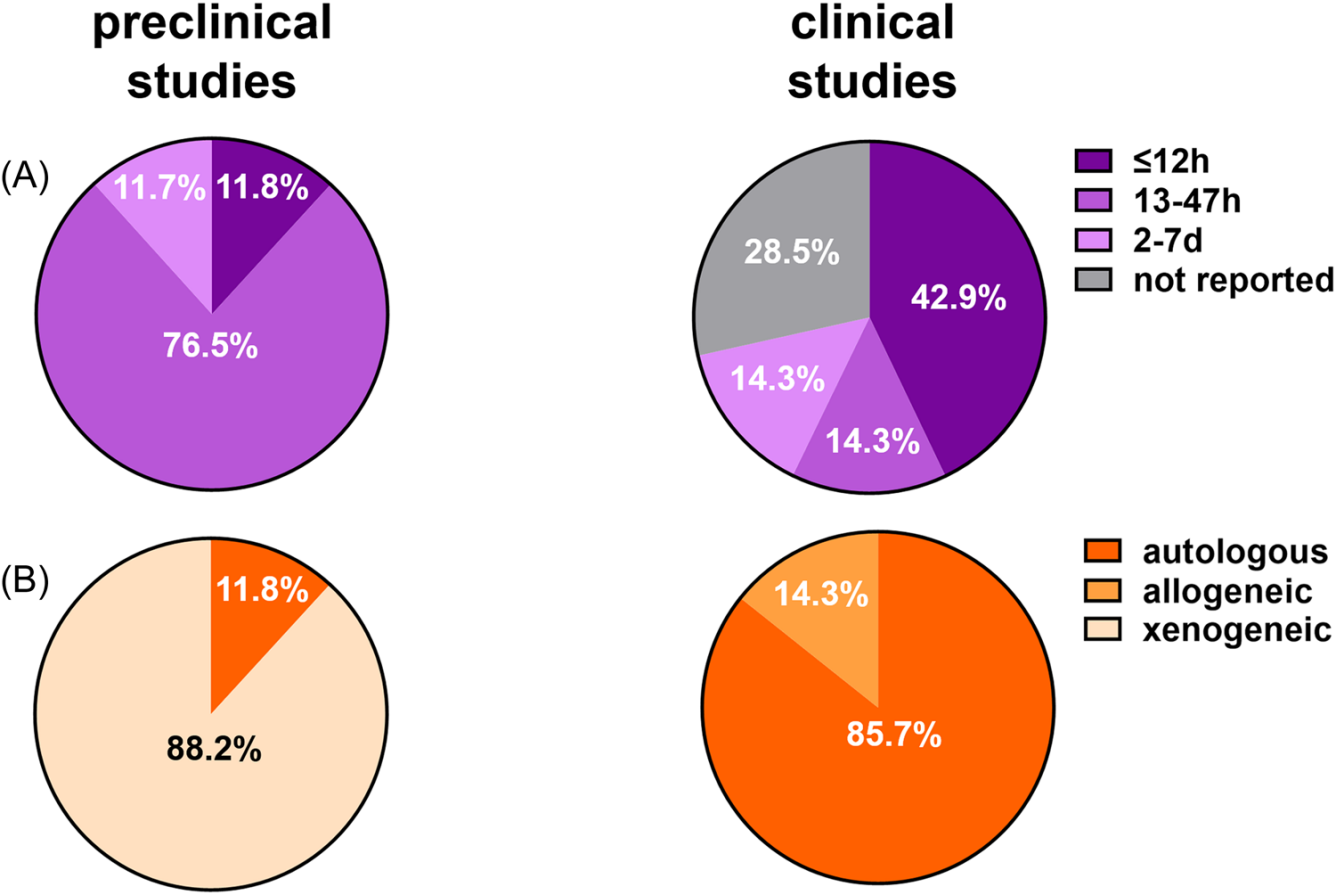
Timing of administration and MNC origin. (A) The proportion of studies administering MNC within the first 12 h after HIE, between 13 and 47 h after HIE, and 2–7 days after the event. (B) The proportion of studies using autologous versus allogeneic and xenogeneic MNC. HIE, hypoxic‐ischemic encephalopathy; MNC, mononuclear cell.

The timing of MNC administration was slightly different in preclinical versus clinical studies. MNC were administered within the first 12 h after HIE onset in 11.8% (*n* = 2) of preclinical and in 42.9% (*n* = 3) of clinical studies. Of note, in 28.6% (*n* = 2) of these clinical studies, MNC (from autologous cord blood) was administered immediately, which was not reported for any of the preclinical trials. MNC delivery was performed between 13 and 47 h after HIE onset in 76.5% (*n* = 13) of the preclinical but only in 14.3% (*n* = 1) of the clinical trials. MNC transplantation within 2–7 days after HIE was reported in 11.7% (*n* = 2) of the preclinical and 14.3% (*n* = 1) of the clinical studies. 28.5% (*n* = 2) of clinical studies did not specify the time of MNC administration (Figure 7A).

There were differences in MNC origin between preclinical and clinical studies. 88.2% (*n* = 15) of preclinical studies used xenogeneic MNC; the remaining preclinical studies relied on autologous MNC. Only 1 (14.3%) clinical study used allogenic MNC. Instead, clinical trials predominantly used autologous MNC, reported in 85.7% (*n* = 6) of the trials (Figure 7B). Overall, we have identified extensive design differences between preclinical and clinical trials. These differences may provide an explanation for the downturn in efficacy observed in the transition to clinical trials.

#### 3.7.4 Study endpoints

There were clear differences in the type of primary endpoints addressed between the preclinical and clinical studies. Efficacy of treatment was the primary outcome used in 100% of the preclinical studies; 64.7% (*n* = 11) measured functional outcomes, and 35.3% (*n* = 6) measured brain histochemical parameters. In contrast, for all but one of the clinical studies (85.7%, *n* = 6), safety/mortality was defined as the primary endpoint. Analysis of blood parameters was the primary endpoint of the remaining clinical trial (14.3%).

## 4 DISCUSSION

This systematic review and meta‐analysis aimed to provide potential explanations as to why a clear therapeutic efficacy may not have been observed in clinical studies using MNC as an experimental treatment for HIE. Obviously, the clinical trials using MNC conducted so far were early‐stage clinical trials addressing safety and mortality as the primary endpoint. They may not have been powered to reliably detect the efficacy of MNC treatment for HIE. This may well explain why no clear indication of efficacy was seen in these clinical studies. However, there are also other aspects that may contribute to this.

### 4.1 There is no indication of a general lack of MNC efficacy from preclinical HIE studies

A potential reason may be that the MNC is not potent enough to provide therapeutic benefits after a global cerebral hypoxic‐ischemic event with a potentially catastrophic impact on brain function and outcome. However, MNCs were clearly efficient in all preclinical studies analyzed (Figure 2A). Although some studies reported extreme effect sizes, a leave‐one‐out‐analyses did not indicate that the overall clear efficacy of MNC treatments in HIE animal models may be caused by single studies reporting particularly good therapeutic outcomes. Thus, there is no strong rationale supported by the overall efficacy data to question MNC efficacy in HIE, at least in animal models.

### 4.2 MNCs were effective independently from preclinical study design parameters what is likely related to the small number of preclinical studies

On the other hand, the efficacy of MNCs did not depend on a single preclinical study design parameter we investigated (Figure 3), suggesting that MNCs were therapeutically effective regardless of how they were applied. However, such an interpretation must be taken with much care as it may not be fully justified for several reasons. First, the number of preclinical studies included was very small while the overall scatter of reported effect sizes was high. This could have “statistically masked” real‐world differences due to high SD and often minimal sizes of category groups analyzed. Second, there are some interesting findings when looking at mean values only. For instance, the largest mean effect sizes were reported after intracerebroventricular MNC administration. Indeed, intracerebroventricular administration is particularly effective in transplanting the largest amounts of cells to the injured brain tissue. Intracerebroventricular administration may also allow good distribution of MNCs throughout the brain tissue, which may be crucial as HIE is a global cerebral event with often widespread damage. Again, the overall number of studies is very small (*n* = 2), making such interpretations purely speculative for the time being. Third, some of the data is not perfectly intuitive. For instance, the average effect size was larger when transplanting less than 5 million cells as compared to transplanting more than 5 million cells. This may, however, be related to the fact that the two studies with the highest effect sizes^26^, ^27^ both used less than 5 million cells. Overall, there was a formal, statistically insignificant weak positive correlation between cell number and effect size (*r*(15) = 0.091; *p* = 0.73; data not shown). If, as commonly believed, MNC predominantly acts via bystander effects,^7^ then a larger number of MNC should be assumed to be more beneficial, unless they are blocking a major blood vessel or ventricle after systemic and intracerebroventricular administration respectively. Indeed, a dose‐dependent beneficial effect of cord blood MNC has been reported after stroke.^42^ Although 30–50 million cells per animal were not more beneficial than 10 million cells, this was more likely related to a ceiling effect rather than a detrimental impact of very high cell numbers. Taken together, there is clearly a need for more preclinical research in the field as the currently available data does not allow for many meaningful conclusions. However, there are also some reassuring observations. MNC cryopreservation before administration did not compromise the cells' therapeutic effect. The number of studies using cryopreserved (*n* = 10) versus freshly prepared MNC (*n* = 7) was relatively well balanced. This finding is important because cryopreservation was previously reported to compromise the beneficial effects of cord blood MNC after ischemic stroke,^43^ so it is reassuring that the situation is potentially different after HIE.

### 4.3 Preclinical study quality is not optimal but not substandard

The formal effectivity of MNC regardless of preclinical study design parameters may, in theory, be explained by low study quality. Indeed, the low methodological quality of a study may increase the risk of bias and therefore an (often severe) overestimation of therapeutic effect size as evidenced in the related field of ischemic stroke.^44^, ^45^ Employing a quality score based on the STAIR and STEPS guidelines, we found that there were some shortcomings in overall methodological study quality (Figure 4).

Interestingly, the frequency with which relevant quality assurance measures are applied in experimental studies in cardiovascular and related fields has recently been investigated.^46^ Of note, the field of stroke is meanwhile leading (but not yet completely sufficient) regarding methodological quality according to Ramirez et al., potentially due to the impact of the 2009 STAIR guidelines.^23^ Ramirez et al. focused on relevant items such as randomization, blinding, and a priori sample size calculation. 70.6% of the studies included in our analysis reported randomization. This frequency is higher than in the field of stroke (45.6%) in which randomization was much more frequently reported than in all other areas investigated by Ramirez et al. (21.5%). The picture is similar to blinding. 76.5% of the HIE preclinical studies reported blinded outcome assessment which is higher than for stroke (62.6%) and other areas (38.3%) according to the data of Ramirez and coworkers. Finally, 5.9% of the HIE preclinical studies reported a priori sample size calculation, compared to 9.5% of preclinical stroke studies and 1.1% in preclinical studies on other conditions. This illustrates that the methodological quality of preclinical studies on HIE is not optimal, but also not worse than in related fields.

The methodological quality regarding the statistical design of preclinical studies was poor in studies focusing on HIE (Figure 4A,B). Unfortunately, no comparable data from other fields are available to the best of our knowledge. Nevertheless, applying improper statistical tests such as parametric tests on nonnormally distributed data or not correcting for multiple testing increases the chances of false‐positive test results. Given the overall suboptimal but not substandard methodological quality of the preclinical HIE studies analyzed, it is unlikely that the poor statistical design quality alone may explain a bias strong enough to induce a massive overestimation of therapeutic effect sizes, making preclinical study results unpredictive of the clinical situation.

### 4.4 Relevant design differences in preclinical versus clinical studies using MNC in HIE

There were, however, relevant differences in preclinical versus clinical study design. For instance, there were differences in study subject ages. Human patients, relative to their time of birth, were consistently younger than animals included in preclinical studies, but given the higher maturity at birth of humans as compared to the rodents (94.1% rats, 5.9% mice) used in preclinical studies, the impact of this difference is unclear.

A striking difference was that 100% of the clinical studies applied TH, whereas none of the preclinical studies did so. Thus, preclinical and clinical studies compare an experimental MNC treatment against a different control paradigm, namely TH treatment in clinical studies, but the untreated condition in preclinical ones. Although the therapeutic impact of TH regarding functional deficits is modest, any additional therapeutic impact provided by MNC in a clinical setup must be large enough to be statistically discriminable against TH as the control condition. A small but potentially synergistic impact of MNC treatment may be much harder to detect in clinical studies, especially given the higher heterogeneity of cases. Since the use of a confirmed effective treatment, even with modest effects, is imperative in human patients, future preclinical studies on HIE should introduce TH as a control paradigm and combination treatment.

Although more routes of MNC administration were investigated in preclinical studies, 15 out of 17 studies (88.2%) utilized routes that can be considered as “systemic” (intravenous, intraarterial, intraperitoneal) MNC administration. This is basically comparable to the intravenous transplantation route used for MNC administration in all human HIE patients. Intravenous cell transplantation is considered the most feasible and least dangerous route.^14^ Thus, there are no real alternatives to intravenous MNC administration for safety and feasibility reasons, particularly when treating newborns in early‐stage clinical trials. Thus, preclinical trials should rely on intravenous (or at least systemic) transplantation unless therapeutic efficacy of another route of MNC administration against intravenous/systemic administration should be investigated.

The majority of preclinical studies (82.4%) used a fixed cell dose, but most of the clinical that provided respective information used a dose range. This may be related to the fact that mostly autologous UCB MNCs were used in clinical trials (the rest used allogeneic UCB MNCs), so patients were likely administered with the number of MNCs that were available. This is understandable but induces additional heterogeneity in the study population making it more difficult to detect a therapeutic impact of MNC treatment. Moreover, the dosing paradigm should be disclosed in all clinical studies. While clinical studies consistently applied for higher MNC numbers, the situation is different when referring to cell doses. Considering cells/kg is a reasonable approach as systemic administration was predominantly performed in both preclinical and clinical studies. Only two preclinical studies using rats reported the subjects' weight, ranging from 12.1 to 15.3 g. Similar weights can be assumed in other studies as all but one used rats on the seventh postnatal day. When assuming treatment with 10 million MNC, the dose would be between 653.6 and 826.4 million cells/kg. In turn, if assuming newborns weight of approximately 3.5 kg, 50 million cells would translate into just 14.3 million cells/kg. The difference may even be more dramatic when applying a body surface area‐based allometric dose translation. Bystander effects as mediated by MNC are predominantly mediated by beneficial factors acting in a paracrine or endocrine manner, and strong indirect evidence for this has been previously reported.^47^, ^48^, ^49^ Higher MNC doses would increase the amount of these factors being available to the damaged tissue, and it is plausible that this would increase their therapeutic impact. Thus, it cannot be excluded that clinically used MNC doses are too low to induce meaningful therapeutic benefits. Future preclinical studies should adopt clinically realistic MNC doses to investigate this possibility.

There were also differences in experimental MNC treatment timing. More than three out of four preclinical studies transplanted cells within 13 and 47 h after HIE onset, but only one out of seven clinical studies did so. Although the timing was not reported in three clinical studies, the remaining ones performed transplantation within the first 12 h after HIE. Comprehensive data is not available for HIE, but MNCs were reported to have a therapeutic time window of 3–7 days after stroke in rodents.^11^, ^12^, ^50^ However, very early transplantation (3 h after stroke) was reported to be most beneficial in a preclinical study investigating very early transplantation time points.^51^ Although only BM MNC was used in the latter study, there is no strong evidence that very early treatment time points used in clinical HIE studies may be suboptimal. In general, preclinical studies should use transplantation time points that are used clinically, that is, predominantly relying on cell transplantation within the first 12 h after HIE onset.

Nominal differences in study subject age and their potential impact on outcome results require detailed investigation. Newborn rodents and humans are at different developmental stages, so consideration of the developmental age potentially more important than referring to the timepoint of birth. However, not much specific information is available about how exactly rodent and human developmental stages at the time point of birth compare. Potentially, the use of higher mammalian species (i.e., large animal models) may be a way to address these issues. The developmental stage between human and, for instance, ovine or canine newborns is more comparable although not identical.

### 4.5 Limitations

Our study has several limitations. The most obvious one is the relatively small number of studies being included. This made statistical analyses challenging, especially when subcategories were analyzed, further reducing the sample size. Thus, some of the result interpretations, though plausible, remain speculative. Future research and additional original studies are required to overcome this limitation.

Another limitation is that this systematic review and meta‐analysis exclusively focused on MNC. Other cell populations such as MSC or neuronal stem cells have been investigated as potential treatments in HIE for more than a decade.^52^, ^53^, ^54^ Therapeutic use of MSC even seems to be optimized by TH, and both treatments may work synergistically.^55^ However, clinical studies on MSC have only been reported very recently.^56^, ^57^ Thus, no reliable comparison of preclinical and clinical study features would have been possible. With the number of studies using other cell populations, in particular MSC, increasing in the future, investigations like ours should be repeated to gain a more comprehensive picture.

Finally, there is only a very small number of large animal models HIE studies on cell therapies,^58^ and none on MNC. Large animal models became crucial as a validating tool of experimental approaches in stroke, traumatic brain injury, and other conditions^59^, ^60^, ^61^ as their gyrencephalic brain structure, larger brain volumes, higher white matter content, and richer behavioral repertoire are closer to the human situation. This is relevant for the evaluation of experimental therapies including cell‐based approaches.^62^ Moreover, studies using autologous MNC, specifically from UCB, may only be possible in large animal HIE models. Comparing data from those studies, if it was available, would have substantially strengthened our analysis.

## 5 CONCLUSIONS

In summary, there is no indication of a lack of therapeutic MNC efficacy in HIE, at least based on the preclinical data. Preclinical study design, though not optimal at all, was not inferior to other fields of therapeutic research on cardiovascular conditions including stroke. Thus, a massive overestimation of therapeutic effect size in preclinical studies is less likely to explain the lack of convincing indicators of MNC efficacy in clinical studies. However, inherent design requirements in early‐stage clinical studies (small sample size, low statistical power) and, importantly, design differences observed between preclinical and clinical trials may be a plausible explanation. Although definite evidence is lacking, the neglect of TH as a baseline therapy in preclinical studies, the much higher doses of MNC with respect to body weight in preclinical studies, and the higher flexibility in cell doses in clinical trials may be decisive aspects. Future preclinical studies should focus on TH and lower cell doses whereas cell doses applied in clinical studies may be more strictly defined. The use of large animal HIE models shall be considered more frequently in translational research.

## AUTHOR CONTRIBUTIONS

**Alexander M. Scrutton**: data collection; data analysis and discussion; drafting the manuscript; formal analysis; investigation; methodology; validation; visualization; writing— original draft; writing—review and editing. **Francesca Ollis**: Development of the search strategy; data collection and data analysis; formal analysis; investigation; validation; writing—editing and review. **Johannes Boltze**: development of the search strategy; data analysis; drafting the manuscript; conceptualization; formal analysis; methodology; supervision; validation; visualization; writing—original draft and writing—review and editing.

## CONFLICT OF INTEREST STATEMENT

Johannes Boltze is the Editor‐in‐Chief of *Neuroprotection*. He is therefore excluded from the peer‐review process and all editorial decisions related to the publication of this manuscript. The remaining authors declare no conflict of interest.

## ETHICS STATEMENT

No ethical statement is required as this was a systematic review and meta‐analysis based on publicly available data.

## Supporting information

Supporting information.

## Data Availability

Data are available from the corresponding author upon reasonable request.
