# Supplementary material for: Mononuclear cell therapy of neonatal hypoxic‐ischemic encephalopathy in preclinical versus clinical studies: A systematic analysis of therapeutic efficacy and study design
Source: Neuroprotection. 2023 Dec 30;1(2):143–59. doi: 10.1002/nep3.29 (PMC7615506; doi:10.1002/nep3.29)
Supplement: Supplementary file 1 — Supporting information. [file NEP3-1-143-s001.docx]

**Supplementary tables**

**Supplementary table 1. Search strategy for preclinical studies (PubMed, Web of Science, Scopus**)

| **Specification** | **Condition** | **Cells** | **Species** |
| --- | --- | --- | --- |
| neonatal | global cerebral ischemia | bone marrow | mouse |
| perinatal | cerebral ischemia | mononuclear cells | rat |
|  | hypoxic-ischemic encephalopathy | MNC | murine |
|  | HIE | BM-MNC | gerbil |
|  |  | BMMNC | hamster |
|  |  | umbilical cord | rabbit / hare |
|  |  | umbilical cord blood | sheep / lamb / ovine |
|  |  | UCB | goat |
|  |  | UCBC | dog / canine |
|  |  | Wharton jelly | cat / feline |
|  |  | umbilical matrix | pig / swine / porcine |
|  |  |  | non-human primate / NHP / macaque |

Search terms within each column were connected with the Boolean logical operator OR and all columns were connected with the logical operator AND.

**Supplementary table 2. Search strategy for clinical studies (PubMed, Web of Science, Scopus, and clinicaltrials.gov**)

| **Specification** | **Condition** | **Cells** |
| --- | --- | --- |
| neonatal | global cerebral ischemia | bone marrow |
| perinatal | cerebral ischemia | mononuclear cells |
|  | hypoxic-ischemic encephalopathy | MNC |
|  | HIE | BM-MNC |
|  |  | BMMNC |
|  |  | umbilical cord |
|  |  | umbilical cord blood |
|  |  | UCB |
|  |  | UCBC |
|  |  | Wharton jelly |
|  |  | umbilical matrix |

Search terms within each column were connected with the Boolean logical operator OR and all columns were connected with the logical operator AND.

**Supplementary table 3.** **Description of preclinical studies**

| **PMID** | **Authors** | **Year** | **Species / strain** | **HIE model** | **Number of subjects**  **(control / MNC)** | **Age (postnatal)** | **Post-event surveillance** | **Primary endpoint** |
| --- | --- | --- | --- | --- | --- | --- | --- | --- |
| 19430381 | de Paula et al. | 2009 | Rat /  Wistar | Right pCCAO + 8% O_2_ | 10 / 15 | 7 days | 3 weeks post HIE | Functional outcome (Morris water maze) |
| 19296724 | Pimentel-Coelho et al. | 2010 | Rat /  Lister-Hooded | Right pCCAO + 8% O_2_ | 8 / 7 | 7 days | 7 days post HIE | Functional outcome (negative geotaxis test) |
| 22441035 | de Paula et al. | 2012 | Rat /  Wistar | Right pCCAO + 8% O_2_ | 10 / 10 | 7 days | 5 days post transplantation | Functional outcome (Morris water maze) |
| 22526623 | Rosenkranz et al., | 2012 | Rat /  Wistar | Left pCCAO + 8% O_2_ | Not reported | 7 days | 14 days post HIE | Brain histochemistry (caspase-3 expression) |
| 22796290 | Wasielewski et al. | 2012 | Rat /  Wistar | Left pCCAO + 8% O_2_ | 8 / 4 | 7 days | 44 days post HIE | Functional outcome (forelimb asymmetry score) |
| 23123184 | Rosenkranz et al. | 2013 | Rat /  Wistar | Left pCCAO + 8% O_2_ | 4 / 4 | 7 days | 2 days post HIE | Molecular biology (Concentration of interleukin-1-alpha) |
| 23632377 | Wang et al. | 2013 | Rat /  Sprague-Dawley | Left pCCAO + 8% O_2_ | 10 / 10 | 7 days | 14 days post HIE | Brain histochemistry (Neurons/mm^2^) |
| 24177600 | Greggio et al. | 2014 | Rat /  Wistar | Right pCCAO + 8% O_2_ | 9 / 10 | 7 days | 6 days post transplantation | Functional outcome (Morris water maze) |
| 24565927 | Wang et al. | 2014 | Rat /  Sprague-Dawley | Left pCCAO + 8% O_2_ | 20 / 20 | 7 days | 28 days post transplantation | Brain histochemistry (concentration of SHH protein) |
| 25720519 | Hattori et al. | 2015 | Rat /  Wistar | Left pCCAO + 8% O_2_ | 12 / 11 | 7 days | 24 hours post HIE | Brain histochemistry (caspase-3 cells/mm^2^) |
| 28980775 | Grandvuillemin et al. | 2017 | Rat /  Sprague-Dawley | Right pCCAO + 8% O_2_ | 10 / 10 | 7 days | 14 days post HIE | Functional outcone (elevated plus maze) |
| 28281676 | Nakanishi et al. | 2017 | Rat /  Sprague-Dawley | Right pCCAO + 8% O_2_ | 15 /14 | 7 days | 3 weeks post HIE | Functional outcome (cylinder test) |
| 30559704 | Sato et al., | 2018 | Rat /  Wistar | Left pCCAO + 8% O_2_ | 8 / 8 | 7 days | 17 to 18 days post HIE | Functional outcome (Rotarod test) |
| 30967791 | Penny et al., | 2019 | Rat /  Sprague-Dawley | Left pCCAO + 8% O_2_ | 11 / 6 | 7 days | 40 days post HIE | Functional outcome (composite Z-score of negative geotaxis, cylinder test, and open field test) |
| 32157146 | Cho et al. | 2020 | Mouse /  ICR | Right pCCAO + 8% O_2_ | 8 / 8 | 7 days | 35 days post transplantation | Functional outcome (mNSS) |
| 32585139 | Penny et al., | 2020 | Rat /  Sprague-Dawley | Left pCCAO + 8% O_2_ | 30 / 31 | 10 days | 40 days HIE | Functional outcome (composite Z-score of negative geotaxis, cylinder test, and open field test) |
| 35308119 | Lyu et al., | 2022 | Rat /  Not reported | bilateral tCCAO + 8% O_2_ | 12 / 12 | 7 days | 3 months post HIE | Functional outcome (Beam walk test) |

ICR: Institute for Cancer Research; mNSS: modified neurological severity score; p/tCCAO: permanent/transient common carotid artery occlusion; SHH: sonic hedgehoc; VEGF: Vascular endothelial growth factor

**Supplementary table 4.** **Description of preclinical studies**

| **NCT number** | **Last update year** | **Patients** | **Age** | **Post-event surveillance** | **Primary endpoint** | **Secondary endpoints** |
| --- | --- | --- | --- | --- | --- | --- |
| NCT00593242 | 2017 | 52 | Up to 14 days | Not specified | Safety | Unspecified neurodevelopmental function |
| NCT01649648 | 2017 | 2 | 1-3 days | 3 days postnatal | Safety | Functional outcome (Bayley score) |
| NCT02256618 | 2019 | 6 | Up to 24 hours | Not specified | Safety | Functional outcome (Kyoto Scale of Psychological Development) |
| NCT02455830 | 2019 | 18 | Up to 24 hours | 10 days postnatal | Blood analysis of cytokines and trophic factors | Neuroimaging and neurodevelopmental functional outcomes |
| NCT02612155 | 2020 | 35 | Up to 6 hours | Not specified | Safety | Functional outcome (Bayley score) |
| NCT02881970 | 2022 | 20 | 1-3 days | 2 years post treatment | Safety | Unspecified neurodevelopmental function |
| NCT02551003 | 2023 | 60 | Up to 24 hours | 18 months postnatal | Mortality | Functional outcome (Bayley score) |
